# 4D CT image acquisition errors in SBRT of liver identified using correlation

**DOI:** 10.1120/jacmp.v13i1.3564

**Published:** 2012-01-05

**Authors:** Martin Szegedi, Vikren Sarkar, Prema Rassiah‐Szegedi, Brian Wang, Y.Jessica Huang, Hui Zhao, Bill Salter

**Affiliations:** ^1^ Department of Radiation Oncology University of Utah Salt Lake City UT USA

**Keywords:** 4D CT, patient breathing, liver motion, internal fiducials

## Abstract

In the AAPM Report 80,^(^ ^1^ ^)^ the imaging modality of 4D CT and respiration‐correlated CT was declared a “promising solution for obtaining high‐quality CT data in the presence of respiratory motion”. To gather anatomically correct data over time, the existence of correlation between the internal organ movement and an external surrogate has to be assumed. For the in‐house evaluation of such correlation, we retrospectively analyzed 21 four‐dimensional computer tomography (4D CT) scans of five patients, out of which the artifacts experienced in three patients are shown here. To provide context and a baseline for the analysis of patient motion, a real‐tissue liver phantom was used with a solid water block and liver tissue. The superior–inferior motion of fiducials in phantom and patients was correlated to the recorded anterior–posterior motion of an external surrogate marker on the chest. The use of a solid water block yielded a measurable correlation coefficient of 0.98 or better using a sinusoidal animation pattern. With sinusoidally‐animated liver tissue, the minimum correlation observed was 0.96. Comparing this to retrospective patient data, we found three cases of a change in the correlation coefficient, or simply a low correlation. The source of this low correlation was investigated by careful examination of the breathing traces and the CT‐phase assignments used to reconstruct the datasets. Consequences of nonregular breathing are elaborated on. We demonstrate the impact of wrong phase assignments and missing image information in the 4D CT phase sampling processes. We also show how daily patient‐based correlation analysis can indicate changes in breathing traces, which can be significant enough to decrease, or completely eliminate, previously existing correlation.

PACS numbers: 87.57.‐s, 87.57.Q‐, 87.57.cp, 87.57.N‐, 87.55.Qr

## I. INTRODUCTION

In today's radiation therapy programs, the use of 4D CT is the state‐of‐the‐art solution to detect and address patient breathing‐induced tissue motion. AAPM Report 80^(^ ^1^ ^)^ sees 4D CT and respiration‐correlated CT as a “promising solution for obtaining high quality CT data in the presence of respiratory motion”. The report offers a good current summary of motion in the context of radiation therapy.

One inherent assumption for 4D CT is the existence of correlation between internal organ movement and the external surrogate. Correlation has been reliably shown for phantoms induced with a regular, sinusoidal breathing trace. In patient studies, high‐quality CT data (i.e. correct representation of patient anatomy) is imperative to correctly outline anatomical structures, as well as accurately calculate and display doses. This data can only be obtained if proper correlation exists between the surrogate and various organs in the first place.

Recent studies have focused on the correlation validity of 4D CT information, with Beddar et al.^(^ ^2^ ^)^ showing that this correlation generally exists for a cohort of eight patients, with noted variations over the breathing cycle. It is important to note that the accuracy of the 4D CT data itself, used to “measure” and study the correlation, is vulnerable to errors and artifacts due to irregular breathing patterns.^(^ ^1^ ^,^ ^3^ ^)^ It is reasonable to anticipate that a measured correlation might deteriorate if highly irregular breathing is encountered.

This correlation is critical if gated beams or tumor tracking is used for treatment delivery, where there is a potential of treating the wrong area if the correlation is low or if the imaging system is unable to capture the correlation. If the imaging system is unable to capture the correlation resulting in image artifacts, lesion localization and delineation may be compromised due to inconsistent motion estimation.^(^ ^4^ ^,^ ^5^ ^)^ Furthermore, motion artifacts may reduce the accuracy of deformable image registration between different 4D CT phases, thus frustrating the efforts to quantify the accumulated dose in 4D treatment planning.^(^ ^4^ ^–^ ^7^ ^)^

As part of our clinical process, we independently verified and quantified the correlation between implanted liver fiducials and an external surrogate via direct measurement. During this correlation validation, we discovered three cases where there was a lack of correlation between the external marker and internal fiducials. Upon further investigation, we noticed that the integrity of the 4D CT data was affected in each case due to patient irregular breathing, which was picked up by the lack of correlation as displayed by the imaging system. The purpose of this paper is to present these three unexpected imaging results, in which 4D CT proved unreliable in displaying correct patient anatomy in time. The examples discussed show how nonintuitive it can sometimes be to directly evaluate breathing traces for regularity, and demonstrate the resulting CT artifacts that are a direct consequence of nonregular breathing. To provide more context to the reader, we also present the correlation validation and quantification process in our clinic.

## II. MATERIALS AND METHODS

### A. Clinical process and patient selection

Our clinical SBRT protocol requires one 4D CT for treatment planning purposes and a 4D CT done prior to every SBRT fraction.^(^ ^8^ ^)^ Therefore, for most of the SBRT patients treated at our facility, four to six 4D CT datasets have been acquired over the course of treatment. The data of five uncoached free‐breathing liver cancer patients, representing a total of 21 4D CT datasets, was retrospectively analyzed for this study, which was approved by the university institutional review board (IRB #00048188). All patients reviewed here had at least three surgical clips, gold or carbon fiducials (Civco medical solutions, IA), implanted by an interventional radiologist in the liver tissue surrounding the lesion. Patients were immobilized in a customized full‐body Medical Intelligence BodyFIX system (Medical Intelligence, Schwabmuenchen, Germany), and ball bearings (BBs) were placed on the immobilization device and patient's chest wall to mark the initial setup isocenter. Through the use of a 16‐slice GE CT scanner (GE Medical Systems, Milwaukee, WI), 4D and helical scans, with 2.5 mm slice thickness, were acquired for treatment planning purposes and for position verification prior to each treatment session. According to our clinical scanning protocol, patients were oversampled by at least 0.5 sec (120 kV, scout‐determined mA, 0.5 sec rotation, 0.25 sec between images). The axial field of view (A‐FOV) was either 2 cm (for 1.25 mm slice thickness) or 4 cm (for 2.5 mm slice thickness). Since the potential for irregular breathing and breathing drifts increases with an increase in scan duration, our clinical scanning protocol is set up such that the scan duration is minimized, without compromising on proper phase acquisition. Therefore, in the clinic, 4D CT datasets are acquired within approximately 1 minute, independent of the superior–inferior (SI) length of scan. If a 1.25 mm slice thickness does not accommodate the image limit of the GE CT, it will be changed during setup to 2.5 mm. The 4D raw data is then processed into 10 phase‐binned image sets by the GE Advantage Windows (AW) SIM MD software (GE Medical Systems, Milwaukee, WI). Once phase‐binned, AW expresses the absolute value of the farthest image (in the selected phase) and the selected CT‐phase as the Maximum Phase Sampling Error (MPSE). The 4D image acquisition process for GE CT has been described previously by Pan et al.^(^ ^9^ ^)^ Details of the Varian RPM device have also been published.^(^ ^10^ ^,^ ^11^ ^)^

### B. Correlation measurements between the surrogate, bony anatomy, and fiducials

In order to analyze image sets for motion range and correlation, we recorded the coordinates of all fiducials in each phase‐binned image set, along with stationary reference points for each 4D CT dataset. The geometric center of all landmarks was manually identified by a single observer, and distances and positions between fiducials were calculated. Based on the average and standard deviation of the coordinates of a stationary marker found in all reconstructed CT phases, we evaluated how closely we could pinpoint the center of a fiducial. Two fixed reference points in the phantom and two reference points in the patient, one in bony anatomy and one outside of the patient, were used in the analysis. The bony landmark enabled tracking of baseline shifts of the tumor from fraction to fraction. The movement of implanted fiducials and the shift of the geometric center of the implanted fiducials were measured for each reconstructed phase image set. The AP motion of the RPM block (surrogate) was correlated to the corresponding SI directions of the internal fiducial centroid to find the correlation coefficient, $r^{2}$.

### C. Establishing a correlation baseline for a best case scenario

In order to provide context for the measured correlation of patient liver motion, we need a baseline “best‐case‐scenario” of the measurement system itself. Unfortunately, such data from direct measurement is not currently available in the literature. Therefore, we used a phantom with highly reproducible motion to provide this baseline correlation that is meant to reflect the absolute measureable truth under ideal conditions for the imaging system used, before progressing to nonverifiable patient data measurements. The phantom we used is a deformable porcine liver phantom recently described in literature, which is capable of producing realistic diaphragm‐induced liver motion^(^ ^12^ ^)^ comparable to that of humans.^(^ ^13^ ^)^ Using this phantom with a known correlation between surrogate and target, the correlation and imaging limits of the 4D CT acquisition were identified.

**Figure 1:**
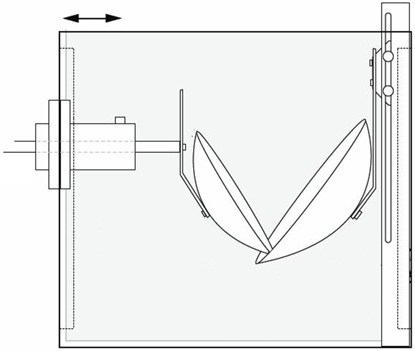
Liver phantom containment. The arrows indicate piston motion direction.

Figure 1 shows a diagram of the phantom used. It contains a diaphragm surrogate that exerts force onto the porcine liver tissue placed between a rigidly fixed support and a moving piston support. To maintain tissue flexibility, Krebs‐Henseleit‐fluid^(^ ^14^ ^)^ surrounds the organ. The phantom's motion characteristics simulate the motion of liver fiducials in real patients.^(^ ^13^ ^,^ ^15^ ^)^ (For more information on the functionality and design of the phantom, the reader is referred to the publication^(^ ^12^ ^)^ of the group that designed it.)

Using this phantom with a rigid object, it can be safely assumed that, under ideal measurement conditions, the measurement will display a perfect correlation. The introduction of deforming tissue in the phantom allows for the assessment of correlation similar to that in patients. For model testing, and to contrast the patient‐related data against an ideal baseline, we used porcine liver with three implanted fiducials with the phantom. We note that no contrast media was used during CT data acquisition for either the phantom or the patients.

Regular sinusoidal (6 sec period, 20 mm peak‐to‐peak SI motion) and irregular patient breathing motion were used to induce motion in the phantom. Using the Varian RPM device (Varian Medical Systems, Palo Alto, CA) to trace motion, we oversampled every axial position (0.5 sec rotation, 0.25 sec between images, 8 sec duration). Repeat 4D scans, with 1.25 mm slice thickness, were acquired with a 16‐slice GE CT. The 4D raw data was processed into 10 separate phase‐binned image sets using the breathing traces acquired with the Varian RPM device.

## III. RESULTS

The values of the fiducials' position in superior–inferior direction are based on increments of slice thickness and, therefore, impose an uncertainty of half the slice dimension. Our error analysis revealed that the standard deviation of fiducial position measurements is submillimeter. We were able to localize the center of a stationary BB from all CT phases with 0.1 mm standard deviation, at 1.25 mm slice thickness with the phantom, and 0.3 mm with 2.5 mm slice thickness in patient scans. Bony landmarks were detected with a standard deviation of 0.2 mm with 1.25 mm slices and 0.3 mm with 2.5 mm slices for all patients. Due to the slice thickness, detection for an individual measurement of liver fiducials in SI direction can be off by up to ±0.6 mm with 1.25 mm slice thickness or up to ±1.25 mm with 2.5 mm slice thickness.

Table 1 displays the overview for the measured patients' motion envelopes. For all fiducials analyzed, we found different motion ranges and direction for every imaging session. MPSE for the patients, as stated by the AW software in percent of GE‐phase sampling, ranged from an average of 3%–4% for well coordinated breathing traces, up to 35% maximum error in one reconstructed phase dataset for irregular and erratic breathing patterns.

**Table 1:** Maximum motion envelope for the marker centroids in all three directions and its summary 3D vector length. The maximum phase error as given by the GE Advantage Windows software is listed in the last column.

	*SI Motion [mm]*	*AP Motion (mm)*	*LR Motion (mm)*	*Sum 3D Vector (mm)*	*MPSE (%)*
Pat. 1	11.9	4.0	2.5	12.8	23
Pat. 2	4.6	2.9	1.3	5.6	13
Pat. 3	10.3	4.4	0.8	11.2	11
Pat. 4	12.5	6.9	4.2	14.9	35
Pat. 5	11.6	7.5	1.3	13.9	26

Table 2 shows the measured motion range of the porcine liver that was animated with a 20 mm piston stroke. With a 8 sec cine sampling per couch position, the MPSE for the phantom was 2% when used with the 6 sec sinusoidal breathing trace, and 4% for an averaged 3.5–4.5 sec period patient trace.

**Table 2:** Maximum motion envelope of the porcine liver for the fiducial centroid in all three directions and its summary 3D vector length. The maximum phase error as given by the GE Advantage Windows software is listed in the last column.

	*SI Motion (mm)*	*AP Motion (mm)*	*LR Motion (mm)*	*Sum 3D Vector (mm)*	*MPSE (%)*
Porcine liver sinusoidal trace	17.2	4.0	1.8	17.7	2
Porcine live patient trace	17.2	4.0	1.8	17.7	4
Rigid object sinuisoidal trace	20	<1	<1	20	2
Rigid object patient trace	20	<1	<1	20	2

Table 3 lists the $r^{2}$ correlation values for the phantom and each of the five patients. The correlation of the phantom piston marker (analogous to the RPM device in use with patients) to the centroid of the three fiducials placed on a rigid object and implanted in the porcine liver was measured twice, with the first measurement for each trace labeled A and the second measurement, taken an hour later, labeled B. The phantoms' sinusoidal animation (SIN) of a rigid object resulted in $r^{2}$ values of 0.999 and 0.98 for two independent measurements. The rigid object animated with a patient trace (PAT) from two independent measurements (A & B) drops the $r^{2}$ values to 0.90 and 0.82.

**Table 3:** Correlation values of the 4D CT internal fiducial in SI direction to external surrogate. Values in bold & italics indicate correlation values of less than or equal to 0.5. The last two columns show correlation data measured with the phantom for a porcine liver and a rigid object.

$r^{2}$ *values of phantom and patients for SI*	*1*	*2*	*3*	*4*	*5*	*Porcine Liver*	*Rigid Object*
Fraction 1	***0.70*** ^*^	0.77	0.92	0.79	0.77	0.99 sin	1.00 sin
Fraction 2	0.97	0.90	0.86	***0.52***	0.91	0.71 pat	0.82 pat
Fraction 3	0.87	0.85	0.94	***0.50***	0.87	0.96 sin	0.98 sin
Fraction 4	0.87	0.80	0.91	0.70	0.69	0.83 pat	0.90 pat
Fraction 5		***0.14***					

* Due to lack of fiducials in one CT phase, this correlation has been calculated with two averaged positions in this CT phase.

**Figure 2:**
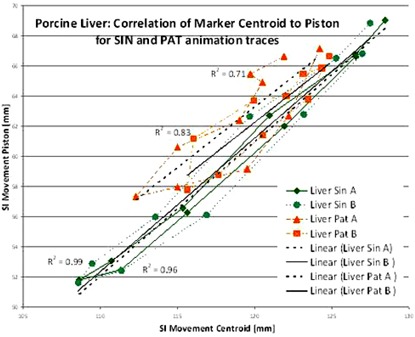
Correlation of the centroid of fiducials in porcine liver to an external marker for SIN‐ and PAT‐animation.

Using a SIN‐animated deforming porcine liver, $r^{2}$ values of 0.99 and 0.96 can be achieved for the correlation of the fiducial centroid with the external surrogate. The same porcine liver animated with the PAT‐trace resulted in $r^{2}$ values of 0.71 and 0.83. The results of the animated porcine liver are shown in Fig. 2.

The patient data showed average to good correlation of the external RPM block with the implanted fiducials' centroid ($r^{2}$ values from 0.50 up to 0.97) when compared to the patient trace animated porcine liver. However, three out of five patients displayed a “RPM to centroid” correlation lower than expected on one or more fractions (displayed in bold and italics in Table 3) or an unusual change of the correlation. Results of further investigation as to why this is and its impact on 4D CT integrity are presented in the discussion section.

## IV. DISCUSSION

### A. Patient fiducial motion range

Apart from patient 2, a very flat breather for whom we have measured the smallest motion envelope of 4.6 mm maximum, all patients show SI motion between 10 mm to 12.5 mm. From Table 1 we can conclude that the patients in our sample fall within the range of motion measured by several other authors.^(^ ^1^ ^,^ ^16^–^27^ ^)^ In more recent studies, such as Suh et al.,^(^ ^17^ ^)^ the reported predominantly linear motion in superior to inferior (SI) direction with a motion range of 0.2 mm to 14.4 mm is comparable to our experience, including the smallest motion envelope of patient 2.

### B. Correlation between fiducial and surrogate

As expected, we found correlation between internal fiducials and an external surrogate, confirming findings by other authors.^(^ ^2^ ^)^ Patient 3 is a good example for correlation with high $r^{2}$ values and so is patient 1. Similarly, patient 2, despite the smallest motion ranges, shows good correlation for four out of five fractions. If correlation values for the multiple fractions are evaluated, one would assume no loss of image integrity or notable artifacts for patient 1, patient 3, and patient 5. This proved to be true for patient 3 and 5, but not patient 1. Detailed analysis of patient 1, 2 and 4 are presented below.

#### B.1 Patient 1 (missing fiducial) analysis

For patient 1, the retrospective attempt to track fiducials via the phase binning process resulted in the “loss” of two fiducials (i.e., two of the three fiducials were not visible in the 0% and 90% resampled 4D CT image sets). Due to this apparent disappearance of fiducials used for motion characterization, the motion envelope of the fiducials and of their centroid, is misrepresented. Similar to patient 4, this error is due to the irregularities of the patients' breathing (i.e.. the limited amount of valid images that were acquired at each couch position). The loss of motion information, with the likelihood of nonrepresented fiducials at the edge of motion envelopes being a worst case scenario, can potentially result in a smaller than needed ITV. The detection of a smaller range of motion can affect planning or image‐guided correctional shifts during treatment.

#### B.2 Patient 2 (one deep breath) analysis

**Figure 3:**
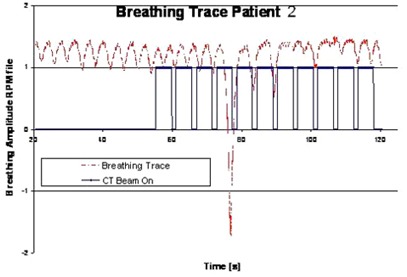
Typical inhale pattern of patient 2 as captured from the RPM device from the 4D CT acquired for fraction 2. The trace shows the total time of image generation.

Patient 2 illustrates the case of a very low measured correlation for the last fraction despite generally good regular breathing performance during four prior fractions showing a low MPSE of 4% to 5%. Upon closer inspection, we found the related RPM breathing traces exhibit a small AP amplitude of only 1.9 mm peak to peak (measured from CT images) with variations and minor irregularities. During the acquisition of the last fraction's 4D CT, the patient exhibited a single deep inhale breathing motion at the fourth axial couch position, shown in Fig. 3. This resulted in a complete set of images being unavailable for that specific axial couch position. Consequently, the phase sorting algorithm replaced the lacking image slices with images in the same position and as close in assigned phase as possible, resulting in MPSEs of 5% and 13% in two out of ten phase sampled CT sets. However, due to the method by which phase is assigned, the end result was a real phase error of up to 50% (i.e., inhale maximums were actually assigned to be exhale maximum).

One of the three fiducials happened to be within the fourth axial couch positioning range where the deep breath had taken place and was assigned to the exhale phase instead of inhale phase. The correlation value, $r^{2}$, of the fiducial centroid to the external surrogate had dropped to 0.14, as seen in Table 3.

Based on the previous four 4D CT scans of this patient (see Table 3), we believe that correlation between the external surrogate and internal fiducials exists, but the imaging system was unable to capture it due to the reason mentioned above. Clinically, this could be damaging for gated patients where the beam‐on time intervals could be mainly in the previously determined (from the simulation CT dataset) beam‐off time slots, thus potentially radiating soft tissue or critical structures and underdosing the PTV.

#### B.3 Patient 4 (drift in breathing) analysis

**Figure 4:**
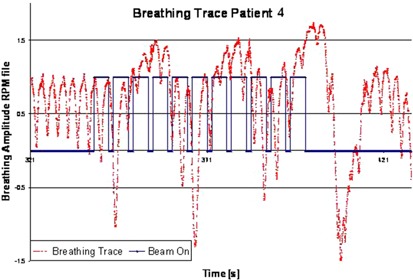
Typical inhale pattern of patient 4 as captured from the RPM device from the 4D CT acquired for the last fraction. The trace shows the total time of image generation.

This patient on first observation displayed the most irregular breathing pattern in our sample. The breathing trace is marked by a slow drift over 6–7 breaths, similar to a SIN wave. An example subset of patient 4's trace is shown in Fig. 4. The initial part of this low frequency SIN wave exhibited progressively deeper inhalation up to a peak inhalation, which also corresponded with the peak of the slow SIN wave. From this point onwards, breathing drifted downwards again with progressively lower individual breaths to the lowest point of the overall SIN curve.

Due to irregular amplitude, several images of the 4D CT were declared invalid by the AW software, leaving the algorithm with fewer images per couch position. This breathing pattern thus challenged the phase‐sorting process with a resulting MPSE of 35%. This shows up in the patients' lower correlation value in general. For two out of four instances (see Table 3), however, wrong RPM phase assignment during fraction 2 and 3 affected one breathing period each, resulting in the images for two couch positions being corrupted and, thus, adding an error similar to that of patient 2 — albeit not as clearly visible through correlation. In general, patient data with such characteristic is prone to anatomical misrepresentation due to the varying motion amplitude, even after phase validation.

### C. General observations

Our clinical protocol does not coach patients' breathing for 4D CT. The resulting broad spectrum of regular and irregular breathing traces and ensuing potential for errors shows a need to improve on the 4D image acquisition technique, specifically when using CT‐based phase‐binning methods.

Beddar et al.^(^ ^2^ ^)^ reduced the probability of MSPE by selectively applying in‐house software for some breathing traces, to better identify CT image phase from the RPM trace. However, the conclusion of that study asserts that fiducial motion is generally well‐correlated with respiratory motion of the RPM device without any data on how commercially available phase assignment systems compare. Suh et al.,^(^ ^17^ ^)^ using the CyberKnife, rebuilt the predictive model when the error in predicted fiducials position in orthogonal images, as a function of surrogate position, was greater than 3 mm. Therefore, their analysis was deemed to be accurate to this level. It should be noted, that the authors did not report how frequently stereo pair images were programmed to occur during treatment, nor did they report on the regularity of the patients' breathing cycles. Irregular breathing is known to induce significant imaging artifacts, which would likely undermine observation of good correlation. Suh et al. reported “good” correlation between surrogate signal and markers. We assume that their patients' predictive data were fairly regular, and that irregularities were not taken into account by the analysis. Neither authors quantified the correlation directly.

While we have been able to show good correlation in patients, it has been shown that seemingly small disturbances in the recording of the breathing trace can have large impacts on the 4D information gained from such a compromised scan. If breathing traces are irregular, it results in phase assignment errors and large MPSEs; therefore, it is important to improve on the image acquisition and image processing in 4D CT. Ideally CT image acquisition would be controlled, in a manner similar to the CyberKnife study^(^ ^17^ ^)^ such that, if predicted motion positions do not compare to measured positions, image acquisition is halted and a new predictive breathing model is generated.

## V. CONCLUSIONS

Today's radiotherapy planning process relies on the accurate determination of motion and deformation during simulation. The impact of wrong phase assignments and missing image information in phase sampling processes, casts doubt onto the correct outlining of target contours, which are needed in treatment planning.

We found comparable liver motion using implanted fiducials as detected by other authors using organ outlines or other methods.^(^ ^21^ ^–^ ^26^ ^)^ Using a phantom produced baseline, the ideal regular breathing case creates ideal correlation between internal fiducials and external surrogate. It was also found that the evaluation of daily patient‐based correlation can indicate changes in breathing trace, which can be significant enough to decrease, or completely eliminate, accurate representation of the anatomy by 4D CT. All three case examples introduced have been detected due to an unusual change of observed correlation. Each of these cases need different adaptive measures to improve on the treatment planning or delivery, the discussion of which is outside the scope of this study.

Future work needs to address clinically improved phase sampling for patients — ideally a predictive tracking system which controls image acquisition, thereby reducing unnecessary dose to the patient while maximizing correct phase assignment.

## ACKNOWLEDGMENTS

The authors would like to thank and acknowledge the grant support from the Department of Radiation Oncology at the Huntsman Cancer Hospital at the University of Utah Healthcare through the Lucille Stohl Kimball Willey Memorial Endowment Fund.
